# Comparative Study of Postmortem MRI and Pathological Findings in Malignant Brain Tumors

**DOI:** 10.7759/cureus.56241

**Published:** 2024-03-15

**Authors:** Norihiko Saito, Nozomi Hirai, Yuki Koyahara, Sho Sato, Yu Hiramoto, Satoshi Fujita, Haruo Nakayama, Morito Hayashi, Keisuke Ito, Satoshi Iwabuchi

**Affiliations:** 1 Neurosurgery, Toho University Ohashi Medical Center, Tokyo, JPN

**Keywords:** t2-weighted image, invasion, autopsy, brain tumor, postmortem mri

## Abstract

This study compared magnetic resonance imaging (MRI) findings of postmortem brain specimens with neuropathological findings to evaluate the value of postmortem MRI. Postmortem MRI was performed on five formalin-fixed whole brains with malignant tumors. Postmortem T2-weighted images detected all neuropathological abnormalities as high-signal regions but also showed histological tumor invasion in areas without edema. Tumor lesions with high necrosis and edema showed high signal intensity on T2-weighted images; in three cases, lesion enlargement was detected on the final prenatal imaging and postmortem MRI. Disease progression immediately before death may have contributed to this difference. In conclusion, the correlation between MRI and neuropathological findings facilitates understanding of the mechanisms responsible for MRI abnormalities. Increased free water due to edema, necrosis, and brain tissue injury can explain the increased signal intensity observed on T2-weighted images. Postmortem MRI may contribute to effective pathology by identifying subtle abnormalities prior to brain dissection.

## Introduction

Malignant brain tumors are not epidemiologically common. However, they have a significant prognostic influence and, depending on the disease state, can cause serious brain dysfunction, leading to sequelae. Glioblastoma, a major malignant brain tumor, is one of the most resistant malignant tumors to treatment, with a mean survival time of less than 15 months, although standard treatments have been established [1,2]. In addition, tumor cell features such as high invasive capacity and proliferative ability can cause serious brain dysfunctions, such as paralysis and impaired consciousness. In such intractable tumors, brain specimens obtained at autopsies are useful not only for understanding the pathology of brain tumors but also for elucidating the effects of treatment and its relationship to the primary brain tissue [3]. However, final magnetic resonance imaging (MRI) before death does not accurately reflect the pathology at the time of autopsy because of the time difference between the time of autopsy and the time of final MRI. Neuroimaging could potentially bridge these gaps as it can detect tissue damage in a non-invasive way and is increasingly available in clinical care. Postmortem MRI of the human brain has advantages compared to in vivo imaging in that it allows examination in ways that are not suitable for living subjects, such as the ability to slice postmortem samples immediately after MRI for histological examination. Unfortunately, most repositories either lack postmortem imaging to correlate with histopathological findings or are restricted to healthy brains or a single disorder. Some studies have previously performed MRI after formalin fixation of autopsy-derived brains. Most involve demyelinating diseases, such as multiple sclerosis and cerebrovascular disorders [4-7], with only a few reports on brain tumors [8,9]. Those reports revealed that T2-weighted images are useful in postmortem MRI, that there is a difference in imaging findings between the final pre-mortem and postmortem MRI, and that tumor invasion is more extensive than inferred from the imaging findings.

However, postmortem imaging of the brain also presents new challenges that have not been addressed by in vivo imaging. In particular, the MRI properties of postmortem tissue can change rapidly as a result of degradation and chemical fixation. These largely unexplored changes in the tissue properties of the postmortem brain may introduce errors in the interpretation of magnetic resonance (MR) findings and complicate the selection of appropriate data acquisition parameters. In this study, we aimed to evaluate the value of postmortem MRI by comparing the MRI findings of postmortem brain specimens with pathological findings in the same section. We believe that this study will contribute to a more detailed understanding of the pathology of the brain.

## Materials and methods

Study setting and participants

This study included five cases of patients with malignant brain tumors treated at our hospital and autopsies performed between 2008 and 2010 at the Toho University Ohashi Medical Center, Tokyo, Japan. Autopsies were performed within 24 hours of death, and the brains were fixed in a 10% buffered formalin solution for at least two weeks. We examined the correspondence between changes in signal intensity on MR images and pathological changes in autopsy brains and between the extent of signal changes and pathological changes.

Standard protocol approvals, registrations, and patient consents

The experimental protocol was approved by the Ethics Committee of Toho University School of Medicine, which did not request informed consent from legal representatives.

Exclusion criteria

Patients without any available medical information were excluded from the study.

MRI protocol and image processing

Before dissection for histopathology, a postmortem MRI of the whole brain, including the cerebral hemispheres, cerebellum, and brainstem up to near the cervicomedullary junction, is performed. All specimens were scanned using a 1.5-tesla MR scanner (Echelon Vega, Hitachi Medical Corporation, Tokyo, Japan). Fixed brains were positioned in a standard manner in the head coil and axial and coronal T2-weighted images. T2-weighted images were acquired in 22 slices of 10 mm thickness, with a display field of view = 24.0 cm × 24.0 cm, repetition time (TR) = 3,500 ms, and echo time (TE) = 96 ms. T1-weighted images showed fewer intensity differences due to the shortening of the relaxation time by formalin fixation. Therefore, T2-weighted images were used in this study.

Pathological examination

The brain was sectioned such that it was in the same cross-sectional plane as the MR image, and large-section specimens were prepared as large-section specimens after gross retrieval. Tissues were stained with hematoxylin and eosin and examined microscopically. The reports of the postmortem MRI and premortem MRI were compared with gross (brain slice) and microscopic findings in five cases. The histology predicted from the postmortem MRI was compared with the final histological diagnosis.

Data collection

Data collected included patient age, gender, pathological diagnosis, and MR images.

## Results

Patient characteristics

The baseline characteristics of the five patients, aged 17-79 (median: 60 years), with malignant brain tumors, are summarized in Table 1. Three patients were male, and two were female. The pathological diagnosis of brain tumors was malignant glioma in three patients and primary central nervous system lymphoma in two patients. The mean duration of formalin fixation was 25.8 days.

**Table 1:** Baseline characteristics for the five patients included in the study

Case no.	Age (years)	Sex	Diagnosis	Formalin fixation duration (days)
1	47	M	Glioblastoma	52
2	17	M	Glioblastoma	17
3	75	F	Glioblastoma	14
4	79	M	Malignant lymphoma	16
5	60	F	Malignant lymphoma	30

Comparison of postmortem MRI and histopathology findings

**Figure 1:**
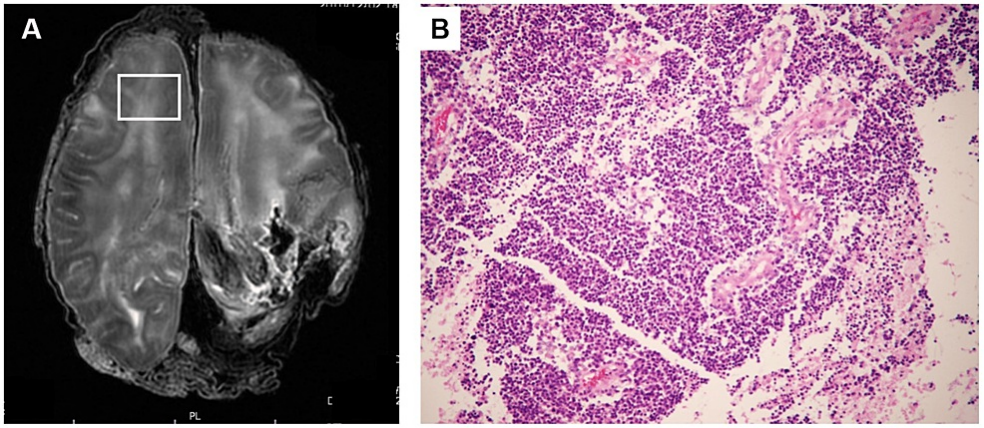
Comparison of postmortem MRI and histopathology findings in a patient diagnosed with glioblastoma (Case 1) A: Postmortem T2-weighted images showing diffuse moderate to high intensities (white box) in the bilateral frontal and temporal white matter. B: Histopathological features of the frontal lobe (white box in Figure 1A) showing highly dense tumor cells without necrosis (hematoxylin and eosin (HE) stain x200).

**Figure 2:**
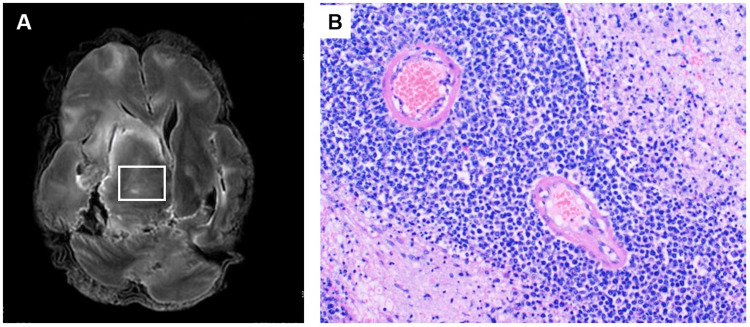
Comparison of postmortem MRI and histopathology findings in a patient diagnosed with primary central nervous system lymphoma (Case 4) A: Postmortem T2-weighted images showing iso-intensity to mildly low intensity in the right basal ganglia (white box). B: Histopathological features of the right basal ganglia (white box in Figure 2A) showing diffuse infiltration of closely packed intermediate to large mononuclear cells with scant cytoplasm (HE stain x200).

The relationship between the pathological findings and areas of signal change on T2-weighted images is shown in Table 2. The tumor tissues showed localized light high-intensity and mild low-intensity signals. A high-intensity signal was observed in three out of five cases and was found to be surrounded by minimal necrosis and edema on histopathology (Figures 1A-1B). Two of the five cases showed iso-to-mildly low intensity, and the tumor tissue was cell-dense with little necrosis or edema on pathological examination (Figures 2A-2B).

**Table 2:** Signal intensity of T2-weighted images and pathological findings in patients diagnosed with a brain tumor

T2-weighted image	Number of cases	Pathological findings
Diffuse moderate high intensity in white matter	5	Edema, radiation necrosis
Focal mild high intensity	5	Tumor
Focal iso-mild low intensity	1	Tumor

**Figure 3:**
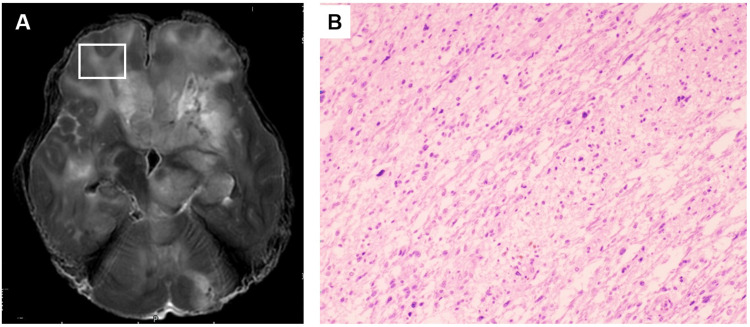
Comparison of postmortem MRI and histopathology findings in a patient diagnosed with glioblastoma (Case 2) A: Postmortem T2-weighted images showing diffuse moderate- to high-intensity signals in the right frontal lobe (white box). B: Histopathological features of the frontal lobe (white box in Figure 3A) showing diffuse infiltration of tumor cells (HE stain x200).

In all five cases, postmortem MRI showed areas of moderately high-intensity signals that appeared to be peritumoral edema; however, pathological examination revealed tumor cells present in these areas. Particularly in glioma cases, extensive tumor cell infiltration was observed (Figures 3A-3B). There were also findings suggesting radiation necrosis; however, it was difficult to distinguish these from brain edema.

Comparison with premortem MRI

**Figure 4:**
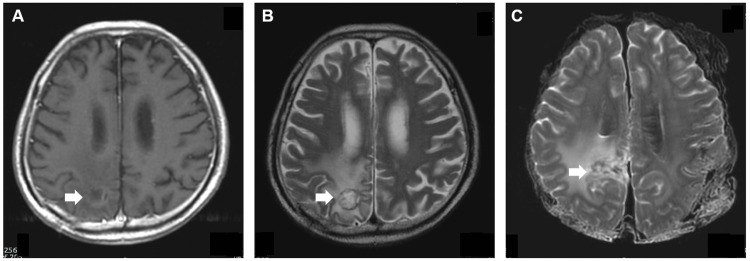
Comparison of the final premortem and postmortem MRI findings in a patient diagnosed with primary central nervous system lymphoma (Case 5) A: T1 contrast image of the final premortem MRI showing a faint contrast-enhanced lesion in the right parietal lobe (arrow). B: T2-weighted image of the final premortem MRI showing high-intensity signals in the right parietal lobe (arrow). C: Postmortem T2-weighted images showing similar findings to the final premortem MRI (arrow).

All patients underwent MRIs, including contrast-enhanced MRIs, within approximately two months prior to death, and in one of the five cases, premortem and autopsy T2-weighted images showed similar findings. The extent of the tumor and brain edema on these images and the pathological findings were generally consistent (Figures 4A-4C).

**Figure 5:**
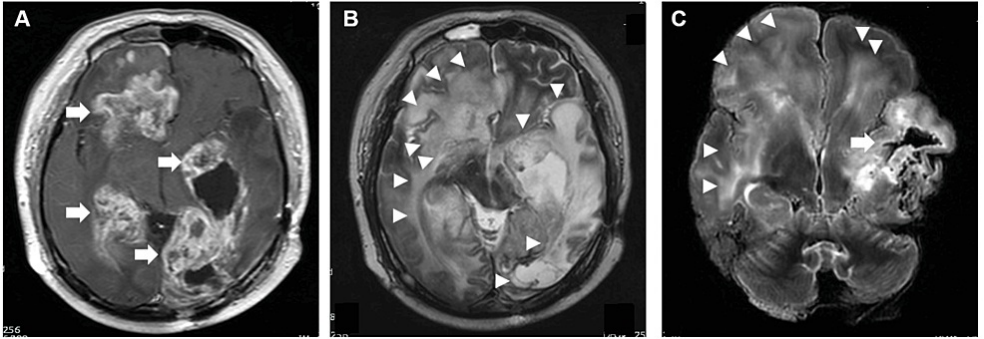
Comparison of the final premortem and postmortem MRI findings in a patient diagnosed with glioblastoma (Case 1) A: T1 contrast image of the final premortem MRI scan showing multiple contrast-enhanced lesions (arrows). B: T2-weighted image of the final premortem MRI showing that T2 high intensity expanded throughout the brain (arrowheads). C: Postmortem T2-weighted images showing T2 high-intensity expansion throughout the brain (arrowheads) and disruptive changes in the brain (arrows).

In four cases, postmortem MRI showed more destructive changes than the premortem MRI due to lesion enlargement and the appearance of new lesions (Figures 5A-5C).

**Figure 6:**
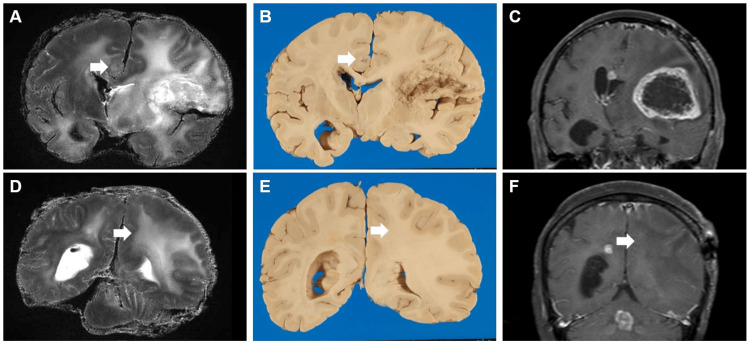
Comparison of premortem and postmortem MRI findings (Case 3) A, B, and C are coronal MRI images of the same cutting plane. A nodular lesion in the right cingulate gyrus with periarcuate in A and B (arrows). A: Postmortem T2-weighted images; B: Brain autopsy; C: T1 contrast image of the final premortem MRI scan. D, E, and F show coronal MRI images of the same cutting plane. T2 high-signal area extension is seen throughout the left hemisphere of the brain (arrows). D: Postmortem T2-weighted images; E: Autopsy of the brain; F: T1 contrast image of the final premortem MRI.

**Figure 7:**
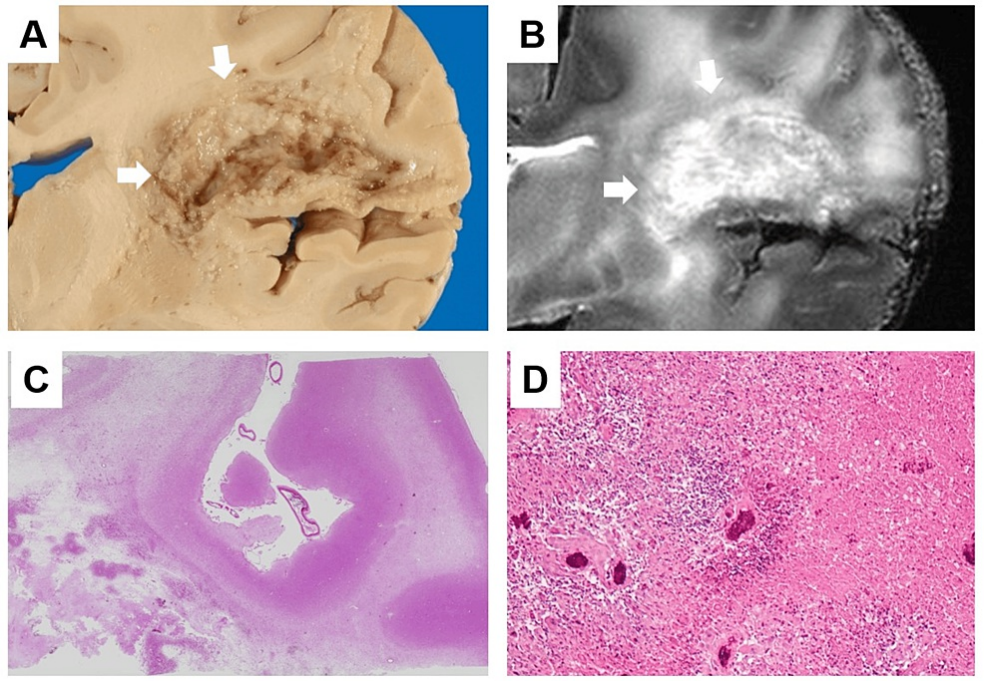
Comparison of brain autopsy, postmortem MRI, and pathology findings (Case 3) A: Macroscopic specimens showing the destructive lesion in the left frontal lobe (arrows). B: Postmortem T2-weighted images showing high intensity in the left frontal lobe (arrows). C, D: Pathological examination revealing extensive necrosis of the left frontal lobe of the cerebrum, the location of the primary lesion, due to radiotherapy (C: HE stain x40, D: HE stain x200).

**Figure 8:**
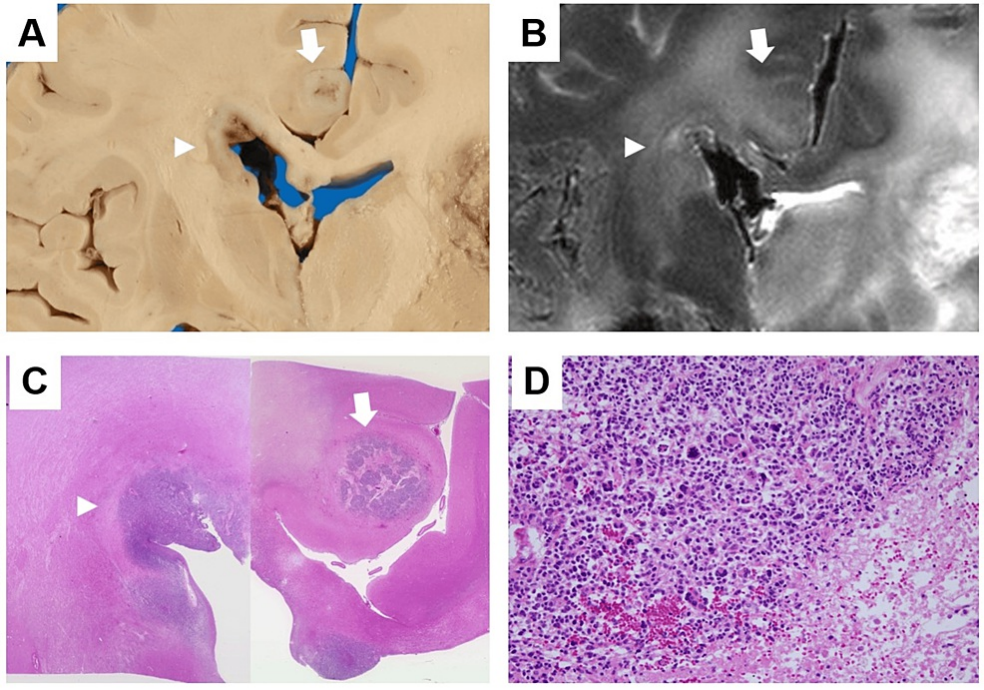
Comparison of brain autopsy, postmortem MRI, and pathology findings (Case 3) A: Macroscopic specimens showing ventricular wall infiltration (arrowhead) and nodular lesions in the cingulate gyrus (arrow). B: Postmortem T2-weighted images showing a high T2-weighted signal with spreading to the ventricular wall (arrowhead) and nodular lesions in the cingulate gyrus (arrow). C: Pathological examination revealing ventricular wall infiltration (arrowhead) and nodular lesions in the cingulate gyrus (arrow) (HE stain x40). D: Pathological examination revealing highly dense tumor cells with necrosis in nodular lesions in the cingulate gyrus (HE stain x200 ).

We present a representative case of a 75-year-old woman (Case 3). The patient died one year and two months after the initial diagnosis of left frontal lobe glioblastoma following surgery, followed by radiation therapy and chemotherapy with temozolomide. An autopsy was performed three hours after death, and the brain was fixed in formalin. Postmortem MRI, performed 14 days after formalin fixation, revealed a left frontal lobe lesion with a high-intensity signal on T2-weighted images and extensive surrounding brain edema. It extended from the corpus callosum to the contralateral hemisphere, and a nodular cluster of tumor cells was observed in the middle of the corpus callosum. A nodular lesion in the right cingulate gyrus with periarcuate brain edema, which was not observed on the final premortem MRI, was also observed (Figures 6A-6F). Pathological examination revealed extensive necrosis of the left frontal lobe of the cerebrum, the location of the primary lesion, after radiotherapy (Figures 7A-7D). In addition, nodular foci of tumor cells were found in the contralateral cerebral hemisphere, lateral ventricles, subarachnoid space, and cerebellum (Figures 8A-8D). Furthermore, diffuse infiltration of tumor cells with large, bizarre nuclei was observed throughout the cerebral hemisphere, brainstem, and cerebellum.

## Discussion

Autopsies are a useful tool for understanding the pathological conditions of deceased patients, even with the development of diagnostic imaging technologies. In particular, malignant brain tumors are highly invasive, and it is necessary to study not only the histopathology of the tumor itself but also its relationship with the surrounding brain as a whole, making brain autopsies highly significant in brain tumor research. Diagnostic imaging is constantly advancing and can provide accurate pictures of pathological conditions. However, there is a time lag between the final imaging examination before death and at death, and the final imaging examination before death may not accurately assess the condition during an autopsy. An MRI examination of the autopsied brain can compensate for this time difference [3,9]. The benefits of postmortem MRI are that it produces a clear image without body motion or pulsating echoes of cerebrospinal fluid and blood, allowing for the comparison of images and pathology in the exact same plane, and does not consider the possibility of lesion progression or change between the time that the MRI is taken and the pathology specimen is examined. Numerous studies have utilized these advantages [10-15]. However, the discrepancy between MRI signal changes in lesions in vivo and autopsy MRI has become a problem [13-15]. Under the imaging conditions we used, the T1-weighted images were unclear and difficult to obtain because of the short T1 relaxation time. However, T2-weighted images showed shortening of both T1 and T2 relaxation times, but the contrast between the signal intensity of the lesion and normal tissue was relatively preserved. It has been reported that abnormal findings on postmortem MR images and premortem MRI are almost identical [13,15]. In this study, the T2-weighted postmortem MRI images in all cases were generally consistent with the premortem images and were also generally consistent with the pathological findings. Therefore, since formalin fixation did not significantly change the MR images, it was meaningful to investigate the correlation between postmortem MR images and pathology. In this study, postmortem MRI was used to identify pathological abnormalities. Tumor lesions showed mild-to-mildly high signal intensity on T2-weighted images. In all cases, signal changes that were more extensive than the site of the neoplastic lesion were associated with edema and radiation necrosis in the same area, whereas those with tumor cells but no signal changes showed no edema or necrosis. Again, the high signal intensity in the T2-weighted images may have been due to the presence or absence of surrounding edema, which had a stronger effect than the distribution of tumor cells [16]. A drawback of postmortem MRI is that contrast studies cannot be performed, and small lesions may potentially be overlooked.

In general, the presence of circulatory disturbances and occupying lesions in the brain, many of which are irreversible, lead to degeneration or necrosis. In such cases, the surrounding tissue tends to become edematous. The edematous areas were more fluid. These lesions also had increased exudates, resulting in increased water content. Increased water content, especially free water, strongly influenced signal changes in the T2-weighted images. High free water content prolongs the T2 relaxation time and produces a strong signal on T2-weighted images [17]. This was confirmed by postmortem MRI.

In this study, four of five cases showed disease progression on postmortem MRI compared with the final premortem findings. This suggests that postmortem MRI may provide useful information for pathology searches. A comparison between premortem MRI and autopsy pathology must always consider the progression and changes in the lesion. However, this is not necessary when comparing postmortem MR images with autopsy pathology, and a detailed examination is possible. Therefore, the results of this study will provide basic knowledge for reading clinical MR images by comparing postmortem MR images with pathological findings.

Limitations of this study

The small sample size may limit the generalizability of our findings and the single-center approach may limit external validity. To increase the robustness and applicability of the conclusions, future studies should consider employing larger and more diverse samples, incorporating control groups, and adopting a multicenter approach. These methods will help ensure that the findings are more generalizable and representative of the population.

## Conclusions

Postmortem MRI showed a generally close correlation between radiological and pathological findings, and the changes caused by formalin fixation of the explanted brain did not significantly affect the MRI readings. Postmortem MRI provides useful information for pathological searches and may be applied to navigational functions.
